# Pharmacist-Physician Communications in a Highly Computerised Hospital: Sign-Off and Action of Electronic Review Messages

**DOI:** 10.1371/journal.pone.0160075

**Published:** 2016-08-09

**Authors:** Sarah K. Pontefract, James Hodson, John F. Marriott, Sabi Redwood, Jamie J. Coleman

**Affiliations:** 1 College of Medical and Dental Sciences, University of Birmingham, Birmingham, United Kingdom; 2 University Hospitals Birmingham NHS Foundation Trust, Edgbaston, Birmingham, United Kingdom; 3 School of Social and Community Medicine,University of Bristol, Bristol, United Kingdom; PreTel Inc, UNITED STATES

## Abstract

**Background:**

Some hospital Computerized Physician Order Entry (CPOE) systems support interprofessional communication. The aim of this study was to investigate the effectiveness of pharmacist-physician messages sent via a CPOE system.

**Method:**

Data from the year 2012 were captured from a large university teaching hospital CPOE database on: 1) review messages assigned by pharmacists; 2) details of the prescription on which the messages were assigned; and 3) details of any changes made to the prescription following a review message being assigned. Data were coded for temporal, message and prescription factors. Messages were analysed to investigate: 1) whether they were signed-off; and 2) the time taken. Messages that requested a measurable action were further analysed to investigate: 1) whether they were actioned as requested; and 2) the time taken. We conducted a multivariable analysis using Generalised Estimating Equations (GEE) to account for the effects of multiple factors simultaneously, and to adjust for any potential correlation between outcomes for repeated review messages on the same prescription. All analyses were performed using SPSS 22 (IBM SPSS Inc., Chicago, IL, USA), with p<0.05 considered significant.

**Results:**

Pharmacists assigned 36,245 review messages to prescriptions over the 12 months, 34,506 of which were coded for analysis after exclusions. Nearly half of messages (46.6%) were signed-off and 65.5% of these were signed-off in ≤ 48 hours. Of the 9,991 further analysed for action, 35.8% led to an action as requested by the pharmacist and just over half of these (57.0%) were actioned in ≤ 24 hours. Factors predictive of an action were the time since the prescription was generated (p<0.001), pharmacist grade (p<0.001), presence of a high-risk medicine (p<0.001), messages relating to reconciliation (p = 0.004), theme of communication (p<0.001), speciality, (p<0.001), category of medicine (p<0.001), and regularity of the prescription (p<0.001).

**Conclusion:**

In this study we observed a lower rate of sign-off and action than we might have expected, suggesting uni-directional communication via the CPOE system may not be optimal. An established pharmacist-physician collaborative working relationship is likely to influence the prioritisation and response to messages, since a more desirable outcome was observed in settings and with grades of pharmacists where this was more likely. Designing systems that can facilitate collaborative communication may be more effective in practice.

## Introduction

The implementation of Electronic Health Records (EHRs) and Computerized Physician Order Entry (CPOE, or electronic prescribing) in hospital provides significant benefits to the quality and safety of patient care [1, 2]. However, the implementation of these technologies has been found to have some unanticipated and unintended consequences, for example, changing patterns of communication between healthcare professionals [3–8]. Since poor or ineffective communication can lead to or contribute to adverse events [9–12], it is important to consider clinician-clinician communication in the design and implementation of these technologies [13].

The medication chart is considered the focal point for physicians, pharmacists and nurses to communicate necessary and relevant medication-related information about a patient. In paper-based prescribing environments, research has shown that pharmacists write on or leave notes on medication charts to facilitate nurse administration or “*subtly influence medical prescribing”* to benefit patient care [14]. This *ad hoc* modality of communication does not require both participants of the communication to be present at the same time and therefore provides flexibility, such as when the message is sent and received. It also helps avoid the need to distract the recipient, with the potential to lead to procedural and clinical errors [15, 16]. Some CPOE systems are designed to facilitate similar interprofessional communication between the pharmacist and physician. However, unlike the paper chart, many CPOE systems also provide information and alerts to help guide decision-making during the medication process, driven by clinical decision support software. This additional “noise” means that the focal point (the medication chart) is no longer just a means of exchanging or gathering information between healthcare professionals. Taking this into account, the effectiveness of clinician-clinician communication via the CPOE system may be influenced by similar factors to those that are believed to influence decision support alerts: namely how and when electronic communications appears to the intended recipient (i.e. passively or alerting); their relevance to the clinician at the time; and how often these are received [17, 18]. In contrast to the paper-chart, CPOE technology also enables clinicians to access and interact with the system from any location, which has the potential to impact on the frequency of interpersonal interactions on the ward [4, 19].

Despite the widespread and increasing use of CPOE systems in hospitals [20, 21], there remains little research into the effectiveness of communications when clinician’s choose to send these electronically, particularly those between the pharmacist and physician [4]. The aim of this study was to investigate the effectiveness of pharmacist-physician communication sent via a CPOE system in a large acute hospital, considering the impact of several temporal, message and prescription factors on the rate of sign-off and action.

## Materials and Methods

### Ethics Statement

The study protocol was approved by the University Hospitals Birmingham NHS Foundation Trust Research and Development Department [21st October 2013] and the University of Birmingham Ethical Review Committee [ERN_12_0127].

### Setting

The Prescribing, Information and Communication System (PICS) is a locally developed CPOE system in use since 2004 at the University Hospital Birmingham NHS Foundation Trust (UHBFT)-a large university teaching hospital (approximately 1300 beds) providing adult acute and elective medical, surgical and specialist care. PICS is used to document the prescribing and administration of medicines throughout all inpatient beds, with the exception of the Emergency Department and some complex systemic anticancer therapies prescribed according to defined treatment protocols. It is also used to generate prescriptions for patients when they are discharged (known as ‘to take out’ prescriptions, or TTOs).

**Fig 1:**
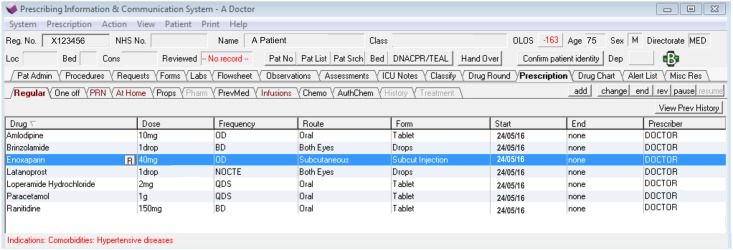
An ‘R’ icon indicates the presence of a review message on prescription order ‘enoxaparin’. Prescription profile generated using PICS Training programme.

**Fig 2:**
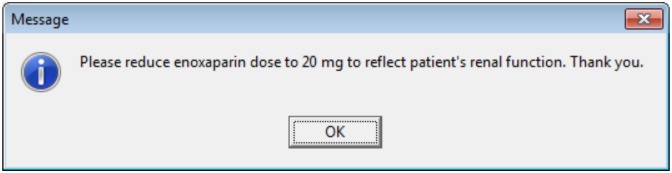
Clicking on the ‘R’ icon reveals the free-text message. Message generated using PICS Training programme.

At UHBFT, pharmacists screen prescription orders that are generated in PICS for their safety and appropriateness. This review is conducted on the ward, although remote working does facilitate review elsewhere when necessary (e.g. in the Pharmacy Department). During review, the pharmacist may wish to query a discrepancy or error, or communicate information to support the order. In PICS, a communication function exists that enables the pharmacist to communicate with the physician using an electronic ‘review message’—a free text message of up to 255 characters that can be assigned to a patient’s prescription. For the purpose of this study, the key features of the review message function are:

1. Delivery of the message is immediate as soon as the pharmacist commits it to the system.
2. An ‘R’ icon identifies the presence of a message (Fig 1). Clicking on the ‘R’ icon reveals the free-text message (Fig 2).
3. The receipt of the message is dependent on when an intended recipient (i.e. physician) next looks at the patient’s prescription profile.
4. The message can be viewed by anyone and is not directed to a named person or team.
5. For each review message, there is an option for the recipient to ‘*sign-off’* the message, which would be considered acknowledgement that the information has been received. Sign-off removes the ‘R’ icon from the prescription. Messages can be signed-off by any healthcare professional. For example, if a prescription has been amended as a result of a request, but the physician failed to sign-off a message, the pharmacist may do so to remove the ‘R’ icon from the screen.
6. The viewing and signing-off of messages is not mandated by the system.

All healthcare professionals involved with the delivery of clinical pharmacy services receive training on the use of PICS as part of their induction. Pharmacists also receive training on the hospital’s clinical pharmacy standards that reflect those stated by the membership body: “*Pharmacists intervene with prescribers*, *patients and other healthcare professionals to ensure medicines are safe and effective*” [22].

### Data capture

The Informatics Department at UHBFT provided data for all review messages assigned by pharmacists to prescriptions between 1^st^ January 2012 and 31^st^ December 2012. For each free-text message captured, information was retrieved relating to the details of the prescription on which it was assigned, whether messages were signed-off within the system, and any changes made to the prescription after the message was assigned (see Table 1).

**Table 1:** Factors included in the generalised estimating equations model.

FACTOR	DESCRIPTION
**Temporal factors**	
Day of the week	Day of the week the review message was assigned by the pharmacist.
Hour of day	Hour of day the review message was assigned by the pharmacist.
Time taken to assign review message	The time between the prescription being generated by the prescriber and the review message being assigned in PICS by the pharmacist.
**Message factors**	
Grade of the pharmacist	Grade of the pharmacist assigning the review message. Grade 6–8; grade 6 pharmacists generally having 0–18 months experience, grade 7 having at least 18 months experience and 8 being the most senior.
High-risk medicine	Messages assigned to high-risk medicines [23].
Medicines reconciliation	Communications relating to a disparity between the patient’s current prescription and the patients medicines on admission.
Communication theme	The subject of the free-text communication (see S1 Appendix).
Profession of person signing off the review message	The profession of the user signing-off the review message (NB: this was analysed for the time taken to sign-off since there is no profession for messages that were not signed-off).
**Prescription factors**	
Speciality	The speciality the patient was under the care of when the review message was assigned.
Category of the medicine	The prescription medicines were categorised according to the chapters of the British National Formulary.
Mode of prescription	The regularity of the prescription (e.g. regular, when required).
Prescription status	D: *Deleted* (the prescription was deleted before any doses were administered). C: *Continued* (the prescription was administered).

### Coding of the data

The captured data were further coded to identify:

1. The theme of communication (see S1 Appendix).
2. Review messages assigned to high-risk medicines [23].
3. Review messages that were not directly associated with the prescription on which they were assigned.
4. Review messages associated with a disparity between what the patient usually takes prior to admission and what they are currently prescribed (i.e. medicines reconciliation).
5. Messages that requested an action that was measureable, and identifiable in the available data.
6. Whether the prescription was changed according to the pharmacist’s request, indicative of an action.

### Outcomes and variables

We considered all factors listed in Table 1, grouped into temporal, message and prescription factors. Four outcomes were considered: 1) sign-off of review messages; 2) whether the time to sign-off was in ≤ 48 hours; 3) action of review messages where a measurable action was recommended; and 4) time to action in ≤ 24 hours.

### Statistical analysis

The outcomes considered in the analysis were split into dichotomous and continuous variables. The continuous variables (time to sign-off and to action) were assessed for normality, and were found to follow a highly skewed distributions that could not be transformed to normality. For this reason, they were dichotomised to create variables indicating whether sign-off occurred in ≤48 hours, and action in ≤24 hours.

We then performed multivariable analyses for the four outcomes in order to consider the associations with a range of factors. The review messages being analysed were not independent since there could be multiple messages on the same prescription. This meant that if a physician signs-off or actions a review message on a patient’s PICS profile, they are likely to sign-off other review messages that exist on the profile at the same time. Hence, the outcomes for repeated messages on a prescription were likely to be correlated. Therefore we analysed the data using multivariable Generalised Estimated Equations (GEE) [24] with binary logistic models and exchangeable correlation structures, which were substituted for unstructured correlation matrices where non-convergence occurred in order to ensure validity. The resulting models found within-prescription correlation ranging from 0.557–0.780 for the outcomes considered, supporting the decision to use the GEE methodology.

Separate models were produced for the two dichotomous outcomes (sign-off and action). For the continuous outcomes (time to sign-off and time to action), the dichotomised versions described previously were used as dependent variables, as valid models could not be produced from non-normal distributed data. Each model contained the temporal, message and prescription factors outlined in Table 1. For any categories where no outcomes occurred, the associated messages were excluded from the respective models, as zero counts made Odds Ratios (ORs) incalculable. In addition, correlations between the factors were assessed to identify potential multicollinearity. This found that ‘high-risk errors’ were highly associated with the route/form theme of communication, so this factor was not included in the analysis. For the analyses of action and time to action, the prescription status (Completed or Deleted) was not considered, since deleting a prescription before it could be administered was one of the behaviours being considered in the definition of an action (i.e. an outcome). All analyses were performed using SPSS 22 (IBM SPSS Inc., Chicago, IL, USA), with p<0.05 considered significant.

## Results

In 2012, 1,291,773 prescriptions were generated for patients in PICS. Pharmacists assigned a total of 36,245 review messages, 34,506 of which were coded for analysis after excluding for messages assigned by other pharmacy staff or that were blank or incomplete (n = 1,739).

**Fig 3:**
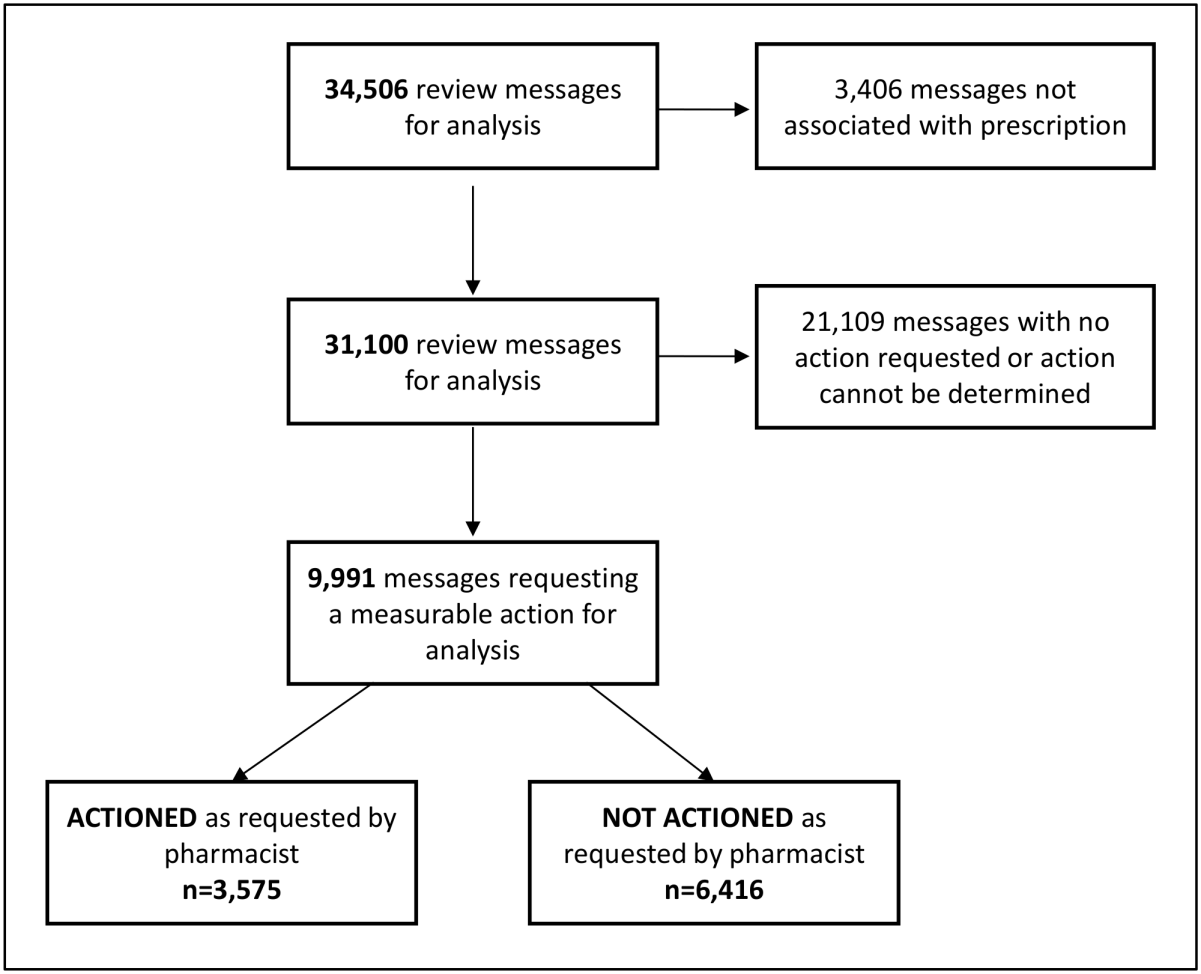
Review messages taken forward for analysis of action.

Out of the 34,506 review messages, 46.6% (n = 16,025/34,506) were signed-off in PICS, the majority of which were by junior doctors (39.5%, n = 6,329/16,025) and pharmacists (39.3%, n = 6,302/16,025). Nearly two thirds of these (65.5%, n = 10,502) were signed-off in ≤ 48 hours. A total of 9,991 review messages across 32 sub-themes were assigned to prescriptions that could be further analysed to determine whether the request had been actioned (Fig 3). Just over a third led to an action as requested by the pharmacist’s communication (35.8%, n = 3,575) and just over half of these (57.0%, n = 2,036) were actioned in ≤ 24 hours.

A second person independently coded approximately 5% (n = 1,722) of the dataset so we could assess inter-rater reliability. This showed substantial agreement across three factors: 1) messages relating to the medicines reconciliation process (97.5%); 2) whether they were associated with the prescription on which they were assigned (99.4%); and 3) the theme of communication (98.8%). A review of the disagreements found no indication of common themes that would imply a systematic error with the coding or require further investigation.

We present the results for each of the temporal, message and prescription factors for the four outcomes: 1) sign-off of review messages; 2) time to sign-off in ≤ 48 hours; 3) action of review messages; and 4) time to action in ≤ 24 hours. The GEE model described accounts for all factors in Table 1 simultaneously. The results tables can be found in supporting information S2–S4 Appendices. Any categories excluded as a result of a zero count are described separately.

### Temporal factors

The results tables of the GEE analysis for temporal factors can be found in S2 Appendix.

#### Day of the week review message assigned

**Fig 4:**
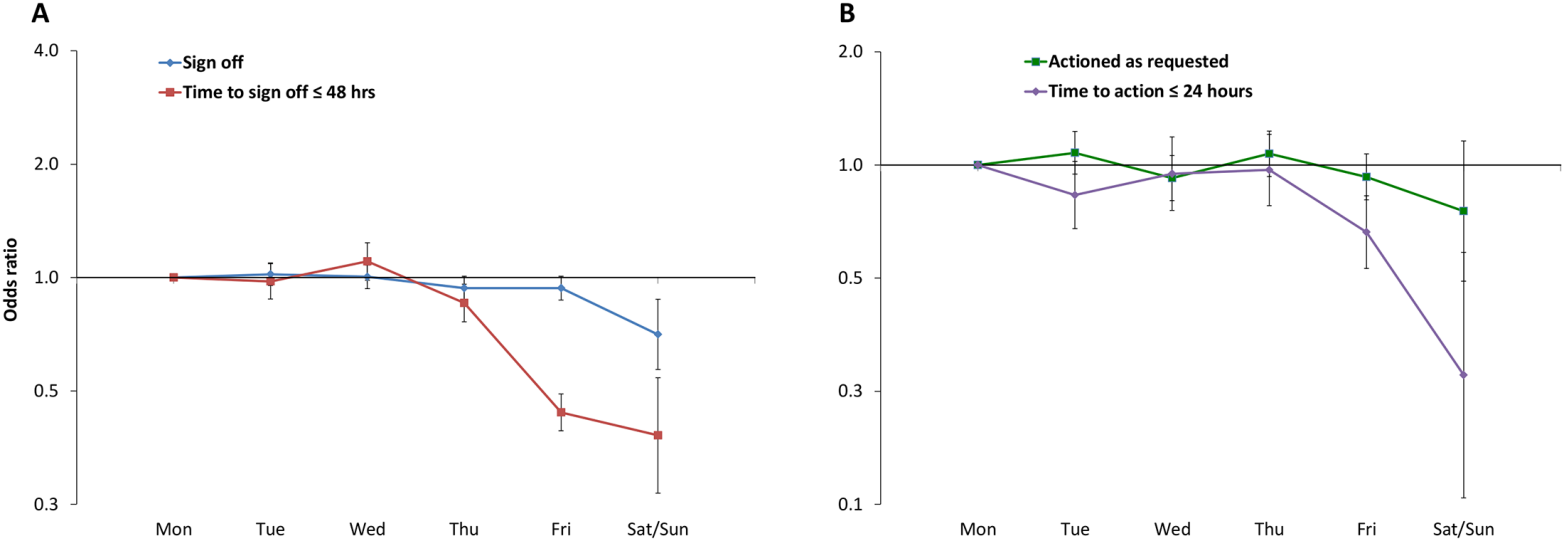
GEE model for day of the week. A: Sign-off and time taken to sign-off in ≤ 48 hours; B: Action and time to action as requested in ≤ 24 hours. ORs (95% CI) from the GEE model described in S2 Appendix. Monday is the reference category and the y-axis uses a logarithmic scale.

The rate of sign-off was significantly different across the days of the week (p = 0.002), with messages less likely to be signed-off at the weekend (p = 0.001, OR 0.706, 95% CI 0.570–0.875) compared to a Monday (Fig 4A). Where messages were signed off, this was significantly less likely to occur in ≤ 48 hours if they were assigned on a Friday (p<0.001, OR 0.439, 95% CI 0.392–0.491) or at the weekend (p<0.001, OR 0.381, 95% CI 0.268–0.542) with median times to sign-off 42.5 hours (range: 1.6–96.5) and 47.1 hours (4.7–72.6) respectively, compared to 23.3 hours (2.4–69.4) on a Monday.

The rate at which messages were actioned did not change significantly across the days of the week (p = 0.073, Fig 4B). However, where messages were actioned, this was less likely to occur in ≤ 24 hours if they were assigned on a Friday (p<0.001, OR 0.663, 95% CI 0.530–0.828) or at the weekend (p = 0.001, OR 0.276, 95% CI 0.130–0.585) relative to a Monday. The median time to action was 22.8 hours (range 1.9–94.1) and 37.3 (10.1–54.1) hours respectively, compared to 20.2 (2.2–48.2) hours on Monday.

#### Hour of day review message is assigned

The hour of day the pharmacist assigned the review messages did not have a significant impact on the rate of sign-off (p = 0.086). However those assigned in the afternoon (13:00–23:59) were less likely to be signed-off in ≤ 48 hours (p = 0.013, OR 0.911, 95% CI 0.846–0.981). No significant difference was detected in the rate of action (p = 0.847) or time to action (p = 0.714) by time of day.

#### Time between prescription being generated and message being assigned

**Fig 5:**
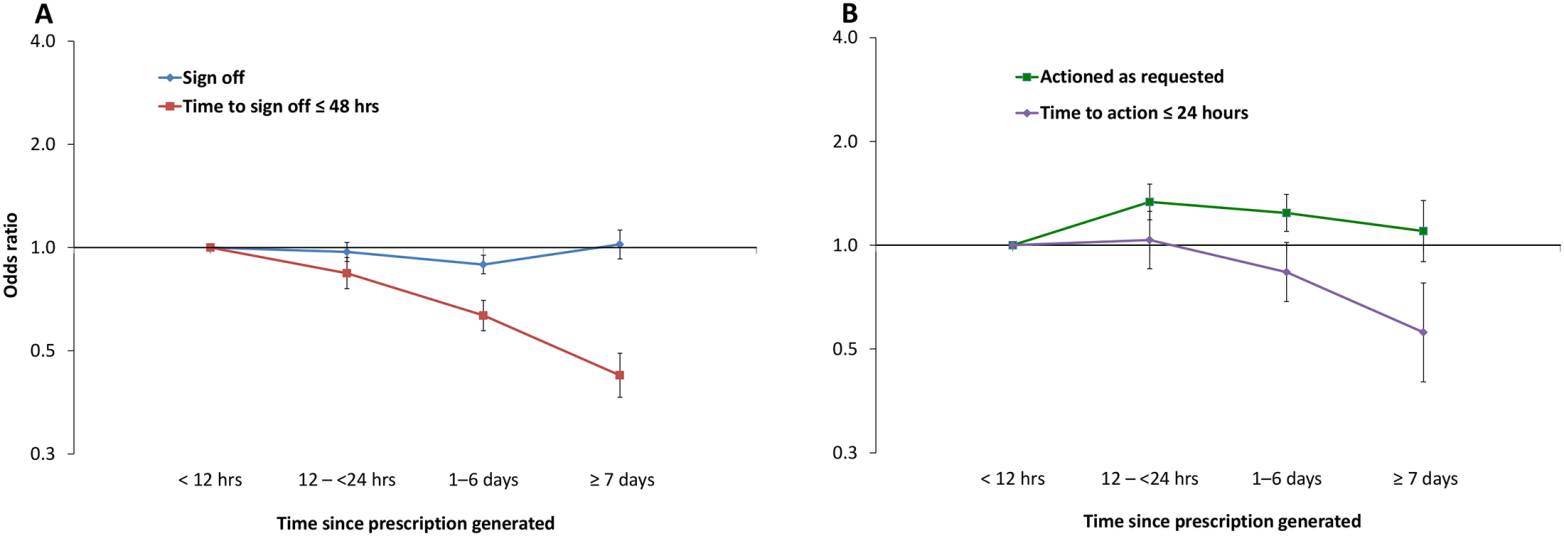
GEE model for the time since the prescription was generated to the message being assigned. A: Sign-off and time to sign-off in ≤ 48 hours; B: Action and time to action as requested in ≤ 24 hours. ORs (95% CI) from the GEE model described in S2 Appendix. < 12 hours is the reference category.

Where messages were signed-off, this was significantly less likely to occur in ≤ 48 hours if they were assigned to prescriptions generated 7 or more days ago, compared to within the last 12 hours (p = 0.001, OR 0.424, 95% CI 0.365–0.492). The former group took on average over twice as long to be signed-off, with a median time of 50.8 hours (range: 12.6–166.6) compared to 20.0 hours (1.2–48.2) (Fig 5A). A similar pattern was observed for those that were actioned, with messages assigned to prescriptions that were generated 7 or more days ago significantly less likely to be actioned in ≤24 hours (p<0.001, OR 0.559, 95% CI 0.402–0.777), taking almost twice as long than those assigned within 12 hours (median 40.3 Vs. 21.6 hours, Fig 5B).

### Message factors

The results tables of the GEE analysis for message factors can be found in S3 Appendix.

#### Grade of pharmacist

The rate of sign-off was significantly different across the grades of the pharmacist (p = 0.010), with those assigned by senior grade 8 pharmacists least likely to be signed-off (p = 0.004, OR 0.899, 95% CI 0.835–0.967 Vs. grade 6). Despite this, it was messages from the grade 8 pharmacists that were the most likely to be actioned (p<0.001, 1.379 95% CI: 1.182–1.607 Vs. grade 6), whilst messages from grade 7 pharmacists were the most likely to be actioned in ≤ 24 hours (p<0.001, OR 1.408, 95% CI: 1.167–1.698).

#### High-risk medicine

Messages assigned to high-risk medicines were significantly less likely to be signed-off (p<0.001, OR 0.841, 95% CI 0.789–0.895) or actioned (p = 0.012, OR 0.848, 95% CI 0.745–0.964) than those assigned to other medicines. However, no significant association was detected between high-risk medicines and the time taken to sign-off or action (p = 0.713 and p = 0.707 respectively).

#### Medicines reconciliation

Messages communicating reconciliation information were significantly more likely to be signed-off (p<0.004, OR 1.082 (1.025–1.142), and then signed-off in ≤ 48 hours (p<0.001, OR 1.210, 95% CI 1.110–1.319) compared to those that were not. These messages were also more likely to be actioned (p<0.001, OR 1.278, 95% CI 1.144–1.428), although the rate of action in ≤ 24 hours was not found to be significantly associated with the communication of reconciliation information (p = 0.859).

#### Communication theme

The theme of communication had a significant impact on whether messages were signed-off (p<0.001). With the exception of communications categorised as Other, all communications were significantly less likely to be signed-off compared to Dose/Frequency, with those related to Contraindication the least likely to be signed-off. Messages relating to a Contraindication were also least likely to be signed-off ≤ 48 hours (p = 0.004, OR 0.721, 95% CI 0.579–0.899 Vs. Dose/Frequency). The rate of action was also found to differ significantly across the categories (p = 0.014), with those relating to ‘Drug Use/Administration’ least likely to be actioned (p = 0.035, OR 0.680, 95% CI 0.475–0.973) Vs. Dose/Frequency).

### Prescription factors

The results tables of the GEE analysis for prescription factors can be found in S4 Appendix.

#### Speciality

**Fig 6:**
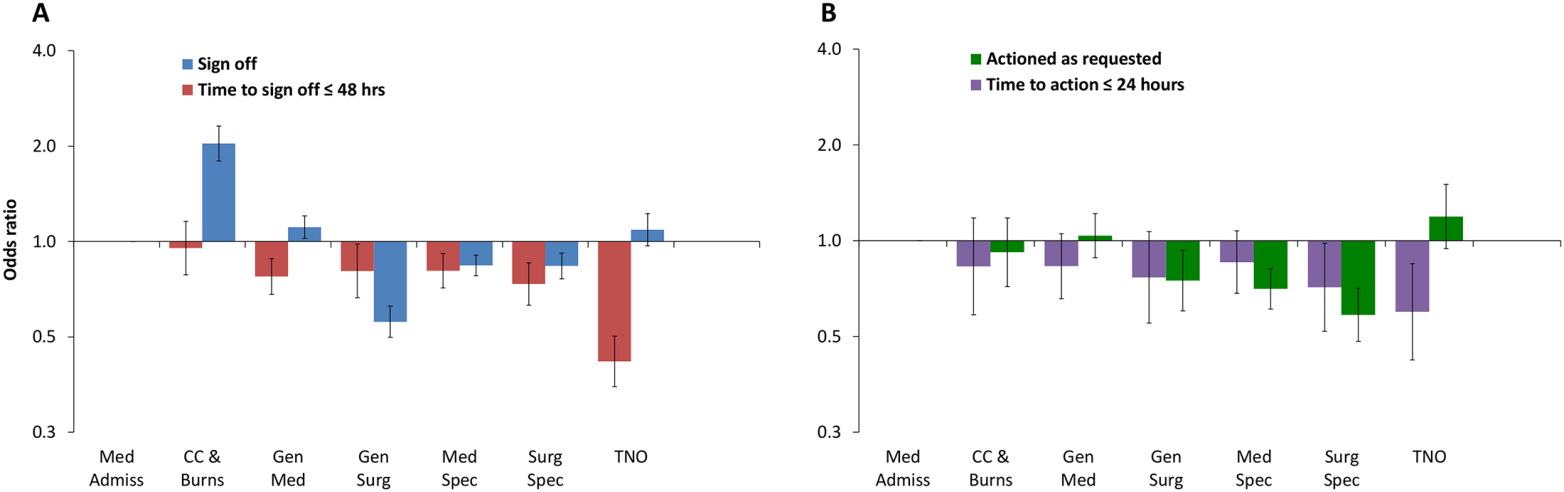
GEE model for speciality. A: Sign-off and time taken to sign-off in ≤ 48 hours; B: Action and time to action as requested in ≤ 24 hours. ORs (95% CI) from the GEE model described in S4 Appendix. Medical Admissions is the reference categoryCC: Critical Care; Med: Medicine; Surg: Surgery; Spec: Specialities; TNO: Trauma and Orthopaedics.

The rate of sign-off (p<0.001) and time taken to sign-off (p<0.001) were found to differ significantly across the specialties (Fig 6A). Where messages were signed-off, this was significantly less likely to occur in ≤ 48 hours if they were assigned to patients in Trauma and Orthopaedics (TNO) than those in Medical Admissions, taking on average over twice as long (p<0.001, OR 0.419, 95% CI 0.349–0.502)—median: 51.3 hours Vs. 20.5 hours. There was a significant difference in the rate of messages being actioned (p<0.001) across the specialities (Fig 6B). Messages were least likely to be actioned in ≤ 24 hours if they were assigned to prescriptions for patients in TNO than in Medical Admissions (p = 0.004, OR 0.598, 95% CI 0.422–0.848), with median times of 28.3 hours (range: 7.2–110.9) compared to 19.9 hours (2.2–36.0).

#### Category of medicine

The rates of sign-off of messages was found to differ across categories (p<0.001). Anti-infective medicines (Infection category) stood out amongst all the categories. Where messages were signed-off, this was twice as likely to occur in ≤ 48 hours (p<0.001, OR 2.062, 95% CI 1.775–0.563), they were significantly more likely to be actioned (p = 0.013, OR 1.246, 95% CI 1.048–1.482), and actioned nearly four times faster than messages assigned to Cardiovascular medicines (p<0.001, OR 2.055, 95% CI 1.546–2.732), with a median time to action of 23.0 Vs. 6.0 hours.

#### Mode of prescription

The mode of the prescription had a significant impact on the rate of sign-off (p<0.001). Where messages were signed-off, this was significantly more likely to occur in ≤ 48 hours if they were assigned to Once-only (p = 0.001, OR 11.077, 95% CI 2.581–47.533) and TTO prescriptions (p<0.001, OR 2.818, 95% CI 2.321–3.421) than ‘Regular’ prescriptions, with a respective median time to sign-off of 1.8 hours (range: 0.1–20.2) and 2.1 hours (0.3–26.2), compared to 23.7 hours (2.5–73.8). As required prescriptions were significantly less likely to be signed-off in ≤ 48 hours (p = 0.004, OR 0.813, 95% CI 0.780–0.935), with a median time of 30.9 hours (3.7–119.9). Since messages on Once-only and TTO prescriptions were less frequent, and rarely specified actions, prescriptions of these types were excluded from the analysis of actions. The analysis found that the messages assigned to ‘As required’ prescriptions were significantly less likely to be actioned than those on Regular prescriptions (p<0.001, OR 0.374, 95% CI 0.317–0.442).

#### Prescription status

Messages assigned to Deleted prescriptions were significantly less likely to be signed-off than those for Completed prescriptions (p = 0.005, OR 0.898, 95% CI 0.832–0.968). However, a higher proportion were signed-off in ≤ 48 hours (p<0.001, OR 1.739, 95% CI 1.520–1.989)—median time of 5.3 hours (0.8–44.1) compared to 24.0 hours (2.5–76.2) for prescriptions that were continued. Prescription status was not included in the analyses of action and time to action, since a deleted prescription was considered an outcome.

## Discussion

We analysed thousands of free-text communications written by pharmacists to physicians and sent via a CPOE system in a large acute hospital. The extensive audit system allowed us to consider several factors that might influence the sign-off and action of messages, and the time taken.

The low rate of sign-off (n = 46.6%) may suggest that this function is not always utilised by the physician as acknowledgment that the information from the pharmacist has been read and/or actioned. Those messages that were analysed for action would be considered interventions by the assigning pharmacist since they requested a “*change in a patient’s management or therapy”* [25]. We observed a lower rate of action (n = 35.8%) than we might have expected compared to other pharmacist intervention studies in the context of CPOE, where acceptance rates have been found to range from 86−90% [26–28]. This may suggest that communication via the CPOE system is sub-optimal at the study site and messages are not being received as intended. Alternatively, messages may have been considered by the physician and a decision made that, in the most part, an action was not necessary [8]. When physicians did choose to sign-off and/or action requests, this did not always occur in a timely manner, with two thirds (65.5%) signed-off in ≤ 48 hours and just over half (57.0%) actioned in ≤ 24 hours. A delay in the acceptance of interventions has previously been observed when they were requested via a CPOE system compared to oral communication [28], which may suggest physicians do not always prioritise medication-related tasks communicated electronically [29]. We believe our findings may be explained by a number of system and process factors, which we discuss below.

### Process factors

It is perhaps not surprising that various time-related factors affected the sign-off and action of messages. Over the weekend when the wards are likely to be staffed by a reduced number of physicians, the rate of sign-off was lower, and the time taken to both sign-off and action messages was found to increase on Fridays and at weekends. A separate study in the same hospital setting found that junior doctors took significantly less time to generate prescriptions in the CPOE system at the weekend [30]. Together with our findings, this may suggest that on-call / covering physicians spend less time interacting with a patient’s prescription profile over the weekend or do not prioritise review messages when services are typically reduced both inside and outside the hospital [31]. The delay could also be explained by the physicians’ lack of confidence to act requests without consulting more senior colleagues, or on behalf of another clinical team. The fact that messages communicated on a Friday also took longer to action may relate to the messages being assigned after the consultant ward rounds (which typically occur in the morning); messages may not be seen until the next routine review of the patient. However, a reduction in the presence of ward pharmacists at the weekend (and perhaps in the afternoon) should not be discounted as a contributing factor here; their presence may act as a visual or verbal prompt for physicians on weekdays to pay attention to medication-related tasks and in a more timely manner.

Typically, the longer a prescription had existed for a patient, the longer it would take for a message to be signed-off or actioned. Physicians may enter a patient’s PICS profile more frequently to monitor response or to optimise treatment to new prescriptions, increasing the opportunity of seeing a message for an action to occur. The pharmacist may also direct their attention to new orders in their prioritisation of tasks, influencing the time to action with additional verbal requests to the physician as they await a response to their message [32]. This finding may highlight a need to encourage regular review of all prescriptions during a patient’s admission.

We found the grade of the pharmacist to be predictive of both action and action in ≤ 24 hours, with physicians less likely to sign-off messages assigned by the highest grade pharmacists, but more likely to action their requests. This factor has previously been found to be a significant predictor of physician acceptance of interventions [33]. In the UK, grade 6 (relatively newly qualified) pharmacists typically rotate every 3 months to gain experience across a range of specialities. As pharmacists move to higher grades, they are more likely to work within a single speciality and to have a more consistent presence on the ward, allowing more time for physicians to better understand and appreciate the knowledge and skills the pharmacist can provide to the team [34]. This is likely to promote collaborative working, which may influence physician response to pharmacist’s requests and how these are prioritised.

High-risk medicines were, perhaps worryingly, less likely to be signed-off or actioned. Although the majority of prescriptions are generated by junior doctors in NHS Hospitals [35], in most cases they are not the decision maker and follow instructions by senior medical colleagues [36, 37]. As such, the physician may be cautious to make changes to a prescription until consulting more senior colleagues, leaving messages on screen for others in the team to view. However, if this were the case, a delay in the time taken for both outcomes would be expected, which we did not observe. In contrast, messages relating to the medicines reconciliation process were more likely to be signed-off and actioned. Given that these requests involve amending a prescription to reflect a patient’s ‘usual’ regimen, the physician is not being asked to make a decision about a new prescription per se. As such, they may perceive these as more straightforward, and without the need to consult more senior colleagues. The difference in the rate of sign-off and action according to the drug category may provide further evidence that physicians find certain requests and medications—such as those relating to cardiovascular drugs—easier to take action on, possibly because of their familiarity with the associated regimens. Messages regarding the Dose /Frequency of medicines were significantly more likely to be signed-off compared to all other types of messages, with the lowest rate observed for contraindicated medicines. This finding did not correlate with the action, suggesting that some requests were not deemed necessary. These findings relating to high-risk medicines, medicines reconciliation and the theme of the message, may all be reflective of the prescribers’ confidence to alter regimens. This may serve as evidence that electronic communication should only be used for non-urgent requests [38], while others require face-to-face or direct (collaborative) discussion between the pharmacist and the physician, which would provide opportunity to gain more context of the patient and the request.

Finally, there was a significant difference in the rate of sign-off and action according to the specialty of the patient. At the study site, the Medical Admissions ward—used as the reference category—has fairly consistent staffing levels across the day and night compared to the on-call cover systems on medical and surgical wards. A pharmacist is present all day Monday–Friday on this ward (as they are for Critical Care and Burns), unlike Surgical Specialities where the pharmacist will visit on a daily basis, but will not be present all day. Physicians are likely to be more familiar with the pharmacist in these settings and have developed a mutual understanding of each other’s expectations with regards medication-related tasks. For example, the pharmacist may actively encourage physicians to engage with the review messages before the end of their shift. A low acceptance rate of interventions has previously been observed in a hospital where the pharmacists communicated entirely via the CPOE system and did not participate in ward rounds [39]. Adopting a process of minimal face-to-face communication would not be considered collaborative, or promote such working. In contrast, a study investigating the impact of CPOE in the “*team-orientated*” Critical Care setting found it did not have a negative impact on the quality of communication in the long-term [40].

### System factors

Review messages in PICS may be considered a type of alert to the physician, albeit a passive one that is non-interruptive. Human factor variables found to influence acceptance of alerts are: 1) display characteristics (i.e. proximity of the alert to the event); 2) textual information; and 3) prioritisation [41]. The sign-off function is intended to serve as an indication that a message has been received, read and acknowledged. However, the rate of sign-off would indicate that it may not be used as intended. A failure to sign-off a message means it remains on a patient’s prescription order, and as such, may unnecessarily contribute to a message burden on screen. This may have an unintended consequence of reducing the effectiveness of messages, with new messages indistinguishable from the old. Messages may become invisible or are no longer obvious to the physician and so may be overlooked [39, 42]. This may provide some explanation as to why a large majority of messages were signed-off by pharmacists themselves (39.3%, n = 6,302/16,025), actively removing the ‘R’ icon from the screen on the doctors’ behalf to ensure any remaining messages still require acknowledgement. This finding suggests that further training is required to promote optimal use of the system—interprofessional sessions would also enable practitioners to share their expectations on the use of the system.

In PICS a pharmacist is unable to assign a priority to a review message to identify those that require more urgent acknowledgment or response. Without this information the physician is unable to appropriately prioritise medication-related requests over other tasks. This may provide some explanation for the observed delay in the sign-off and action of some messages. On the other hand, it was reassuring to find that messages assigned to anti-infective medicines were more likely to be actioned in ≤ 24 hours compared to other medicines. Again this suggests that physicians prioritise the review of some medicines over others; in the particular case of anti-infectives, it may be as a result of national campaigns to raise awareness of antimicrobial stewardship [43].

The review message function does not allow for bi-directional communication and so the physician is not obliged to respond in order to gather information about the query [50]. Therefore, aside from signing-off a message, the physician cannot provide an explanation for their subsequent action (or inaction). Clinical decision support systems that allow clinicians to provide an explanation as to why alerts have been over-ridden have been found to be more likely to succeed than those that did not [44]. Therefore designing systems with bi-directional communication may increase the physician’s awareness of messages, which over time could inform its optimal use. For example, it may reduce the total number of messages assigned by pharmacists as they understand what information is useful to the physician and how best this should be communicated. Two-way communication would also increase collaborative working, which we feel is integral to the effectiveness of this modality of communication.

## Limitations

The aim of this study was to investigate the effectiveness of pharmacist-physician communications when sent via a CPOE system, a modality of communication that is likely to increase as these technologies are introduced into the hospital setting. We did not investigate verbal communication, a modality that evidence suggests results in a timelier acceptance of requests [28] and is likely to be occurring simultaneously.

The language used by pharmacists in their written discourse was not analysed in this study. This may change depending on the grade or prior experience of the pharmacist [14] and could provide further explanation for the outcome of communications. Messages were reviewed and coded to identify the explicit subject, and not the latent content (i.e. what is implied). Latent analysis is not possible without knowing the individual pharmacists and running an analysis of their intent and the subsequent interpretation of the recipient. By its very nature, coding free-text can introduce an element of subjectivity, however the inter-rater reliability study was reassuring and showed that the definitions of the codes were effective and therefore the coding consistent.

In our analysis, we did not identify whether a prescribing error had occurred, since this would have required assumptions to be made of the data without context of the patient or the situation at the time. Therefore only those messages analysed for action can be directly compared to intervention studies. In addition, since this was a retrospective study, we did not interview physicians to determine why requests were not actioned or to explain any time delays.

Finally, this study was conducted in a single-centre, and the results here may not reflect practice in other hospital settings that use electronic communications. We understand that there are likely to be many factors at play to influence the outcomes investigated, and this is being investigated further with focus groups and observational research at the study site.

## Conclusion

The capability to communicate in an *ad hoc* asynchronous manner in hospital has benefits for both the pharmacist and physician—fewer interruptions reduce the need for the physician to multi-task, which can reduce procedural and clinical errors in a busy and pressured environment. However, in this study we observed a lower rate of sign-off and action than we might have expected, suggesting uni-directional communication via the CPOE system may not be optimal. An established pharmacist-physician collaborative working relationship is likely to influence the prioritisation and response to messages, since a more desirable outcome was observed in settings and with grades of pharmacists where this was more likely. Designing systems that can facilitate collaborative communication, such as with the ability for the physician to respond, may be more effective in practice.

## Supporting Information

S1 Appendix Classification of pharmacist-physician communication themes. (DOCX)

S2 Appendix Results of Generalized Estimating Equation (GEE) for temporal factors. (DOCX)

S3 Appendix Results of Generalized Estimating Equation (GEE) for message factors. (DOCX)

S4 Appendix Results of Generalized Estimating Equation (GEE) for prescription factors. (DOCX)

## References

[1] Ranji SR, Rennke S, Wachter RM. Computerised provider order entry combined with clinical decision support systems to improve medication safety: a narrative review. BMJ Quality & Safety. 2014 10.1136/bmjqs-2013-002165 24728888

[2] Nuckols TK, Smith-Spangler C, Morton SC, Asch SM, Patel VM, Anderson LJ, et al The effectiveness of computerized order entry at reducing preventable adverse drug events and medication errors in hospital settings: a systematic review and meta-analysis. Systematic Reviews. 2014;3:56-. 10.1186/2046-4053-3-56. PubMed PMID: PMC4096499. 24894078 PMC4096499

[3] Campbell EM, Sittig DF, Ash JS, Guappone KP, Dykstra RH. Types of unintended consequences related to computerized provider order entry. J Am Med Inform Assoc. 2006;13(5):547–56. Epub 2006/06/27. M2042 [pii] 10.1197/jamia.M2042 16799128 PMC1561794

[4] Thomas SK, Coleman JJ. The impact of computerised physician order entry with integrated clinical decision support on pharmacist–physician communication in the hospital setting: a systematic review of the literature. European Journal of Hospital Pharmacy: Science and Practice. 2012;19(3):349–54. 10.1136/ejhpharm-2012-000110

[5] Georgiou A, Westbrook J, Braithwaite J, Iedema R. Multiple perspectives on the impact of electronic ordering on hospital organisational and communication processes. HIM J. 2006;34(4):130–5. 18216417 10.1177/183335830503400406

[6] Ash JS, Sittig DF, Poon EG, Guappone K, Campbell E, Dykstra RH. The extent and importance of unintended consequences related to computerized provider order entry. J Am Med Inform Assoc. 2007;14(4):415–23. Epub 2007/04/27. M2373 [pii] 10.1197/jamia.M2373 17460127 PMC2244906

[7] Aarts J, Ash J, Berg M. Extending the understanding of computerized physician order entry: implications for professional collaboration, workflow and quality of care. Int J Med Inform. 2007;76 Suppl 1:S4–13. Epub 2006/06/27. S1386-5056(06)00126-2 [pii] 10.1016/j.ijmedinf.2006.05.009 . 16798068

[8] Niazkhani Z, Pirnejad H, van der Sijs H, de Bont A, Aarts J. Computerized provider order entry system—does it support the inter-professional medication process? Lessons from a Dutch academic hospital. Methods Inf Med. 2010;49(1):20–7. Epub 2009/05/19. 0631 [pii] 10.3414/ME0631 . 19448890

[9] Zinn C. 14,000 preventable deaths in Australian hospitals. BMJ. 1995;310:1487. 10.1136/bmj.310.6993.1487 7787586

[10] Ackermann EW. The Quality in Australian Health Care Study. Med J Aust. 1996;164(5):315 Epub 1996/03/04. . 8628171 10.5694/j.1326-5377.1996.tb94205.x

[11] Reason J. Beyond the organisational accident: the need for “error wisdom” on the frontline. Quality and Safety in Health Care. 2004;13(suppl 2):ii28–ii33. 10.1136/qshc.2003.009548 15576688 PMC1765802

[12] The Joint Commission on Accreditation of Healthcare Organizations. Sentinel Events Statistics 2015. Available from: http://www.jointcommission.org/sentinel_event.aspx. 16933568

[13] US Office of the National Coordinator Health Information Technology. Safety Assurance Factors for EHR Resilience Guides 2014 [1 November 2015]. Available from: https://www.healthit.gov/safer/safer-guides.

[14] Liu W, Manias E, Gerdtz M. Medication communication through documentation in medical wards: knowledge and power relations. Nursing Inquiry. 2014;21(3):246–58. 10.1111/nin.12043 23822212

[15] Popovici I, Morita PP, Doran D, Lapinsky S, Morra D, Shier A, et al Technological aspects of hospital communication challenges: an observational study. International Journal for Quality in Health Care. 2015;27(3):183–8. 10.1093/intqhc/mzv016 25855753

[16] Westbrook JI, Coiera E, Dunsmuir WTM, Brown BM, Kelk N, Paoloni R, et al The impact of interruptions on clinical task completion. Quality and Safety in Health Care. 2010;19(4):284–9. 10.1136/qshc.2009.039255 20463369

[17] van der Sijs H, Aarts J, Vulto A, Berg M. Overriding of Drug Safety Alerts in Computerized Physician Order Entry. Journal of the American Medical Informatics Association. 2006;13(2):138–47. 10.1197/jamia.M1809 16357358 PMC1447540

[18] Coleman JJ, van der Sijs H, Haefeli WE, Slight SP, McDowell SE, Seidling HM, et al On the alert: future priorities for alerts in clinical decision support for computerized physician order entry identified from a European workshop. BMC Medical Informatics and Decision Making. 2013;13(1):1–8. 10.1186/1472-6947-13-111 24083548 PMC3850158

[19] Niazkhani Z, Pirnejad H, Berg M, Aarts J. The impact of computerized provider order entry systems on inpatient clinical workflow: a literature review. J Am Med Inform Assoc. 2009;16(4):539–49. Epub 2009/04/25. M2419 [pii] 10.1197/jamia.M2419 19390113 PMC2705258

[20] Ahmed Z, McLeod M, Franklin B, Jacklin A, Barber N. The use and functionality of electronic prescribing systems in English acute NHS trusts: a cross-sectional survey. PLoS ONE. 2013;8:e80378 10.1371/journal.pone.0080378 24278279 PMC3835329

[21] Pedersen CA, Schneider PJ, Scheckelhoff DJ. ASHP national survey of pharmacy practice in hospital settings: Dispensing and administration—2014. American Journal of Health-System Pharmacy. 2015;72(13):1119–37. 10.2146/ajhp150032 26092963

[22] Royal Pharmaceutical Society. Professional Standards for Hospital Pharmacy Services: Optimising patient outcomes from medicines 2014 [11 May 2016]. Available from: http://www.rpharms.com/hospital-pharmacy-services/professional-standards.asp.

[23] Cousins DH, Gerrett D, Warner B. A review of medication incidents reported to the National Reporting and Learning System in England and Wales over six years (2005–2010). British Journal of Clinical Pharmacology. 2012;74(4):597–604. 10.1111/j.1365-2125.2011.04166.x 22188210 PMC3477327

[24] Liang KY, Zeger SL. Longitudinal data analysis using generalized linear models. Biometrika. 1986;73(1):13–22. 10.1093/biomet/73.1.13

[25] Dooley MJ, Allen KM, Doecke CJ, Galbraith KJ, Taylor GR, Bright J, et al A prospective multicentre study of pharmacist initiated changes to drug therapy and patient management in acute care government funded hospitals. Br J Clin Pharmacol. 2004;57(4):513–21. Epub 2004/03/18. 10.1046/j.1365-2125.2003.02029.x BCP2029 [pii]. 15025751 PMC1884463

[26] Bourne R, Choo C. Pharmacist proactive medication recommendations using electronic documentation in a UK general critical care unit. International Journal of Clinical Pharmacy. 2012;34(2):351–7. 10.1007/s11096-012-9613-7 22354852

[27] Ibáñez-Garcia S, Rodriguez-Gonzalez CG, Martin-Barbero ML, Sanjurjo-Saez M, Herranz-Alonso A, iPharma. Adding value through pharmacy validation: a safety and cost perspective. Journal of Evaluation in Clinical Practice. 2016;22(2):253–60. 10.1111/jep.12466 26552362

[28] Bedouch P, Tessier A, Baudrant M, Labarere J, Foroni L, Calop J, et al Computerized physician order entry system combined with on-ward pharmacist: analysis of pharmacists' interventions. J Eval Clin Pract. 2011 Available: http://www.ncbi.nlm.nih.gov/pubmed/21689216 10.1111/j.1365-2753.2011.01704.x 21689216

[29] Brown PJ. What do Physicians Read (and Ignore) in Electronic Progress Notes? Applied Clinical Informatics. 2014;5(2):430–44. 10.4338/ACI-2014-01-RA-0003 25024759 PMC4081746

[30] Coleman JJ, Hodson J, Thomas SK, Brooks HL, Ferner RE. Temporal and other factors that influence the time doctors take to prescribe using an electronic prescribing system 2015 2015-01-01 00:00:00. 206–12 p. 10.1136/amiajnl-2014-002822 PMC4433371 25074989

[31] Freemantle N, Ray D, McNulty D, Rosser D, Bennett S, Keogh BE, et al Increased mortality associated with weekend hospital admission: a case for expanded seven day services? BMJ. 2016;351 10.1136/bmj.h4596 26342923

[32] Wu RC, Lo V, Morra D, Wong BM, Sargeant R, Locke K, et al The intended and unintended consequences of communication systems on general internal medicine inpatient care delivery: a prospective observational case study of five teaching hospitals. J Am Med Inform Assoc. 2013 Epub 2013/01/29. amiajnl-2012-001160 [pii] 10.1136/amiajnl-2012-001160 . 23355461 PMC3721154

[33] Barber N, Batty R, Ridout D. Predicting the rate of physician-accepted interventions by hospital pharmacists in the United Kingdom. American Journal of Health-System Pharmacy. 1997;54(4):397–405. 9043562 10.1093/ajhp/54.4.397

[34] Liu Y, Doucette WR, Farris KB. Examining the development of pharmacist-physician collaboration over 3 months. Res Social Adm Pharm. 2010;6(4):324–33. 10.1016/j.sapharm.2009.11.002 21111389

[35] Lewis PJ, Ashcroft DM, Dornan T, Taylor D, Wass V, Tully MP. Exploring the causes of junior doctors' prescribing mistakes: a qualitative study. British Journal of Clinical Pharmacology. 2014;78(2):310–9. 10.1111/bcp.12332 24517271 PMC4137823

[36] Ross S, Hamilton L, Ryan C, Bond C. Who makes prescribing decisions in hospital inpatients? An observational study. Postgraduate Medical Journal. 2012;88(1043):507–10. 10.1136/postgradmedj-2011-130602 22582180

[37] Dearden E, Mellanby E, Cameron H, Harden J. Which non-technical skills do junior doctors require to prescribe safely? A systematic review. British Journal of Clinical Pharmacology. 2015:n/a–n/a. 10.1111/bcp.12735 PMC4693490 26289988

[38] Edwards A, Fitzpatrick L-A, Augustine S, Trzebucki A, Cheng SL, Presseau C, et al Synchronous communication facilitates interruptive workflow for attending physicians and nurses in clinical settings. International Journal of Medical Informatics. 2009;78(9):629–37. 10.1016/j.ijmedinf.2009.04.006. 10.1016/j.ijmedinf.2009.04.006 19482544

[39] Estellat C, Colombet I, Vautier S, Huault-Quentel J, Durieux P, Sabatier B. Impact of pharmacy validation in a computerized physician order entry context. Int J Qual Health Care. 2007;19(5):317–25. Epub 2007/06/30. mzm025 [pii] 10.1093/intqhc/mzm025 . 17599923

[40] Hoonakker PLT, Carayon P, Walker JM, Brown RL, Cartmill RS. The effects of Computerized Provider Order Entry implementation on communication in Intensive Care Units. International Journal of Medical Informatics. 2013;82(5):e107–17. 10.1016/j.ijmedinf.2012.11.005. 10.1016/j.ijmedinf.2012.11.005 23298435 PMC3624062

[41] Seidling HM, Phansalkar S, Seger DL, Paterno MD, Shaykevich S, Haefeli WE, et al Factors influencing alert acceptance: a novel approach for predicting the success of clinical decision support. Journal of the American Medical Informatics Association. 2011;18(4):479–84. 10.1136/amiajnl-2010-000039 21571746 PMC3128393

[42] Caruba T, Colombet I, Gillaizeau F, Bruni V, Korb V, Prognon P, et al Chronology of prescribing error during the hospital stay and prediction of pharmacist's alerts overriding: a prospective analysis. BMC Health Serv Res. 2010;10:13 Epub 2010/01/14. 1472-6963-10-13 [pii] 10.1186/1472-6963-10-13 20067620 PMC2820036

[43] Department of Health L. Start Smart—Then Focus: Antimicrobial Stewardship Toolkit for English Hospitals In: England PH, editor. London 2015.

[44] Roshanov PS, Fernandes N, Wilczynski JM, Hemens BJ, You JJ, Handler SM, et al Features of effective computerised clinical decision support systems: meta-regression of 162 randomised trials 2013 2013-02-14 14:43:51. 10.1136/bmj.f657 23412440

