# Supplementary material for: Pharmacist-Physician Communications in a Highly Computerised Hospital: Sign-Off and Action of Electronic Review Messages
Source: PLoS One. 2016 Aug 9;11(8):e0160075. doi: 10.1371/journal.pone.0160075 (PMC4978401; doi:10.1371/journal.pone.0160075)
Supplement: S1 Appendix — (DOCX) [file pone.0160075.s001.docx]

### S1 Appendix: Classification of pharmacist-physician communication themes

| **Main category** | **Brief description** | **Examples of medication-related communications** | **Example of pharmacist review message** |
| --- | --- | --- | --- |
| **Contraindication** | *Communication relates to a contraindication to treatment that requires attention or monitoring* | Physiological contraindication | [Bisoprolol] *Patient is currently hypotensive, please do not administer if patient is hypotensive.* |
|  |  | Contraindication exists owing to other disease state | [Nitrofurantoin] e*GFR is less than 60 = please change to trimethoprim.* |
|  |  | Contraindication exists owing to allergy or intolerance | [Trimethoprim] *Patient allergic to co-trimoxazole - contains trimethoprim.* |
|  |  | Potential contraindication as a result of cross-reactivity due to allergy status | [Meropenem] *Patient is allergic to penicillin, if beta lactam allergy, please review and consider alternative.* |
|  |  |  |  |
| **Dose/frequency** | *Communication relates to the dose or frequency of a medicine* | The dose is too high or low for the patient or indication | [Enoxaparin] *Please consider dose reduction to 20 mg daily, patient’s eGFR= 23.* |
|  |  | The wrong dose has been prescribed on conversion of the drug route/form | [Citalopram] *Pls review 20 mg tablet = 16 mg (8 drops)-supplied for NG* [nasogastric] *administration.* |
|  |  | The wrong dose units have been prescribed | [Vancomycin, 1 milligram] *Please amend dose to 1 gram BD.* |
|  |  | The total daily dose has been divided inappropriately for the indication or patient | [Senna] *Pt usually takes ONE BD* [twice a day], *please review.* |
|  |  | The frequency is too high or low for the patient or indication | [Meropenem] *Please review dose of Meropenem, should be BD* [twice a day] *in view of eGFR.* |
|  |  | The regularity of the prescription needs reviewing | [Lactulose, when required] *Most effective when used regularly, please review.* |
|  |  |  |  |
| **Drug form/route** | *Communication relates to the drug form, preparation or route of administration* | Inappropriate drug form for patient or indication | [Lansoprazole capsules] *This patient was prescribed Fastabs prior to admission.* |
|  |  | Inappropriate pharmacokinetic form for patient or indication (e.g. modified-released) | [Propiverine] *This dose is intended to be the XL preparation - please review.* |
|  |  | Inappropriate route for indication or drug | [Chloramphenicol ear drops] *Patient was using chloramphenicol ointment for the right EYE, please review.* |
|  |  | Inappropriate use of multiple routes of administration | [Ranitidine] *Please prescribe IV or oral. Frequency not equivalent (IV must be TDS* [three times a day]*).* |
|  |  | Alternative route recommended for optimal treatment | [Filgrastim] *Please review route due to low platelets should be given IV infusion.* |
|  |  |  |  |
| **Drug interaction** | *Communication relates to a drug interaction that may require attention or monitoring* | Pharmacokinetic drug interaction | [Simvastatin] *Please pause whilst on clarithromycin, increased risk of myopathy.* |
|  |  | Pharmacodynamic drug interaction | [Tramadol] *Increased risk of CNS toxicity when tramadol given with SSRIs. Please monitor.* |
|  |  |  |  |
| **Drug selection** | *Communication relates to the selection of the prescribed drug* | Unsuitable for indication | [Rifampicin] *Micro results show rifampicin resistance - please review appropriateness of this drug.* |
|  |  | Wrong strength of drug/preparation has been prescribed for patient or indication | [Seretide® 250] *Patient uses Seretide 125 evohaler 2 puffs BD - please amend.* |
|  |  | No indication | [Metronidazole] *C diff negative. Is this still needed?* |
|  |  | Use of two drugs with the same active substance | [Paracetamol] *Regular co-codamol prescribed please review prn* [when required] *paracetamol* |
|  |  | Use of two drugs in the same therapeutic group and/or for the same indication | [Lactulose] *Please review use of Laxido and lactulose - therapeutic duplication - both osmotic laxatives* |
|  |  | Sub-optimal drug choice according to guidelines | [Cefotaxime] *First-line treatment for meningitis is ceftriaxone 2 g BD.* |
|  |  | Incorrect product (or drug salt) selected for patient | [Ferrous sulphate] *Pt takes ferrous fumarate 210 mg BD - Please review* |
|  |  | Incorrect drug selected in the electronic prescribing system | [Clonazepam] *Patient does not take clonazepam - see medical notes - patient is prescribed clobazam 20 mg on alternate evenings* |
|  |  |  |  |
| **Drug use/administration process** | *Communication relates to the use of the drug or the administration process* | Timing of administration is inappropriate for the patient, drug or indication | [Dexamethasone] *Please amend timing of doses so that last dose each day is no later than 5pm.* |
|  |  | Drug has not or is not being administered as intended | [Fluoxetine] *Please review - patient refusing doses.* |
|  |  | Patient unable to take/use or be administered drug | [Citalopram] *Dr please review to change to drops as pt* [patient] *having difficulty swallowing.* |
|  |  | Inappropriate drug device for patient or indication | [Seretide Accuhaler®] *Please review to evohaler as per dhx* [drug history]. |
|  |  | Titration regimen or sequential therapy is not prescribed | [Chlordiazepoxide] *Please use the reducing dose regime as per trust guidelines for alcohol withdrawal.* |
|  |  | Patient does not take or use the prescribed drug | [Nicorandil] *Patient no longer takes this, please review.* |
|  |  | Dose is immeasurable for administration | [Enoxaparin] *Treatment dose enoxaparin = 1.5mg/kg 1.5*83 = 124.5 mg - round to 120 mg. Please review dose.* |
|  |  | Duration of prescription is unsuitable or no longer suitable | [Phosphate effervescent] *Please end as PRN phosphate only prescribed on critical care wards.* |
|  |  | Prescription is inappropriate for discharge | [Cyclizine IV] *Please review to tablets for discharge.* |
|  |  |  |  |
| **Logistics** | *Communication relates to the logistics of the medication process* | The prescribed drug is not available for administration | [Calcichew D3 Forte®] *Calcichew D3 forte is non-formulary, please switch to Adcal D3.* |
|  |  | The prescription is incomplete (missing necessary information) | [Fentanyl, TTO] *Needs CD* [controlled drug] *form.* |
|  |  | The prescriber does not have authority to prescribe the drug | [Capecitabine] *Only registrar or consultant to prescribe chemotherapy.* |
|  |  | The prescription is for the wrong patient | [Fentanyl] *To be removed - written for wrong patient.* |
|  |  |  |  |
| **Omission** | *Communication relates to an unintentional omission of treatment* | Omission of a drug taken by a patient prior to admission | [Salbutamol] *Pt also uses Symbicort 400/12.* |
|  |  | Omission of a required drug on discharge prescription | [Metronidazole] *Please add to TTO.* |
|  |  | Omission of a treatment to optimise management | [Adcal D3] *Please note low Hb [haemoglobin] - please could iron supplements be considered.* |
|  |  | Omission of a prophylactic treatment | [TEDS stocking] *Enoxaparin also recommended from thrombosis risk assessment.* |
|  |  |  |  |
| **Supporting information** | *Communication of supporting information or request for supporting information* | Monitoring requirements for treatment | [Amiodarone] *Monitor TFTs* [thyroid function tests]. |
|  |  | Provision of information about the patient that may aid decision-making | [Lansoprazole] *Patient stopped taking this as it was ineffective. He was taking correctly, compliantly and with no drug interactions that would prevent effect.* |
|  |  | Provision of information about the drug that may aid stages of the medication process | [Levetiracetam] *Consider increasing after 1-2 weeks to 250 mg twice daily as per BNF dosing.* |
|  |  | Other supporting information | [Enoxaparin] *Low platelets please review.* |
|  |  |  |  |
| **Other** | *Communication relating to other medication-related issue that is not covered by the other categorises* |  | [.aprepitant] *Aprepitant is in the PICS dictionary; please do not create a new drug.* |
