# Supplementary material for: Pharmacist-Physician Communications in a Highly Computerised Hospital: Sign-Off and Action of Electronic Review Messages
Source: PLoS One. 2016 Aug 9;11(8):e0160075. doi: 10.1371/journal.pone.0160075 (PMC4978401; doi:10.1371/journal.pone.0160075)
Supplement: S2 Appendix — (DOCX) [file pone.0160075.s002.docx]

**S2 Appendix:** Results of Generalized Estimating Equation (GEE) for temporal factors.

**Table A: GEE results for temporal factors for sign-off rates and time to sign-off ≤ 48 hours.**

|  | | **GEE of Sign-off Rates** | | **GEE of Time to Sign-off ≤ 48 hours** | | | **% of Messages** | **Hours to Sign-off**  **(Median, Range)** |
| --- | --- | --- | --- | --- | --- | --- | --- | --- |
|  | | ***Odds Ratio (95% CI)*** | ***p-value*** | ***Odds Ratio (CI)*** | | ***p-value*** | **Signed-off** |  |
| **Day of the week review message assigned** | | | **0.002*** |  |  | **<0.001*** |  |  |
|  | ***Monday*** | 1 | – | 1 | | – | 47.0% | 23.2 (2.4–69.4) |
|  | ***Tuesday*** | 1.020 (0.953–1.093) | 0.563 | 0.977 (0.877–1.088) | | 0.670 | 47.4% | 23.3 (2.7–65.7) |
|  | ***Wednesday*** | 1.004 (0.935–1.079) | 0.903 | 1.104 (0.984–1.237) | | 0.091 | 47.4% | 23.1 (2.2–52.7) |
|  | ***Thursday*** | 0.937 (0.871–1.008) | 0.081 | 0.856 (0.763–0.961) | | 0.008***** | 45.3% | 22.6 (2.0–96.1) |
|  | ***Friday*** | 0.937 (0.872–1.008) | 0.082 | 0.439 (0.392–0.491) | | <0.001* | 45.4% | 42.5 (1.6–96.5) |
|  | ***Sat/Sun*** | 0.706 (0.570–0.875) | 0.001* | 0.381 (0.268–0.542) | | <0.001* | 36.6% | 47.1 (4.7–72.6) |
| **Hour of day review message assigned** | | | **0.086** |  |  | **0.013*** |  |  |
|  | ***00:00–12:59*** | 1 | – | 1 | | – | 46.7% | 23.3 (2.1–72.8) |
|  | ***13:00–23:59*** | 1.043 (0.994–1.093) | 0.086 | 0.911 (0.846–0.981) | | 0.013* | 46.1% | 23.5 (2.6–89.5) |
| **Time from prescription generated to message assigned** | | | **<0.001*** |  |  | **<0.001*** |  |  |
|  | ***< 12 hours*** | 1 | – | 1 | | – | 45.6% | 20.0 (1.2–48.2) |
|  | ***12–23:59 hours*** | 0.971 (0.911–1.035) | 0.367 | 0.841 (0.758–0.934) | | 0.001* | 47.6% | 22.4 (2.0–70.8) |
|  | ***1–6 days*** | 0.892 (0.837–0.949) | <0.001* | 0.633 (0.572–0.701) | | <0.001* | 46.0% | 25.5 (3.1–92.9) |
|  | ***7+ days*** | 1.020 (0.926–1.124) | 0.682 | 0.424 (0.365–0.492) | | 0.001***** | 47.2% | 50.8 (12.6–166.6) |

**Significant at p<0.05*

*Results from GEEs accounting for all factors in Table 1.*

*SIGN-OFF: Profession of person signing off the message was excluded from the analysis since the profession of unsigned messages is not possible to determine.*

**Table B: GEE results for temporal factors for action rates and time taken to action ≤ 24 hours**

|  | | **GEE of Action Rates** | | **GEE of Time to Action ≤ 24 hours** | | | **% of Messages** | **Hours to Action**  **(Median, Range)** |
| --- | --- | --- | --- | --- | --- | --- | --- | --- |
|  | | ***Odds Ratio (95% CI)*** | ***p-value*** | ***Odds Ratio (CI)*** | | ***p-value*** | **Actioned** |  |
| **Day of the week review message assigned** | | | **0.073** |  |  | **<0.001*** |  |  |
|  | ***Monday*** | 1 | – | 1 | | – | 35.7% | 20.2 (2.2–48.2) |
|  | ***Tuesday*** | 1.078 (0.945–1.229) | 0.263 | 0.831 (0.676-1.022) | | 0.079 | 37.8% | 22.1 (2.4–47.3) |
|  | ***Wednesday*** | 0.923 (0.804–1.060) | 0.256 | 0.948 (0.757-1.187) | | 0.640 | 34.6% | 22.3 (2.4–48.0) |
|  | ***Thursday*** | 1.071 (0.932–1.231) | 0.332 | 0.970 (0.779-1.208) | | 0.785 | 36.9% | 21.4 (2.3–45.4) |
|  | ***Friday*** | 0.930 (0.808–1.070) | 0.309 | 0.663 (0.530-0.828) | | <0.001* | 34.2% | 22.8 (1.9–94.1) |
|  | ***Sat/Sun*** | 0.754 (0.490–1.158) | 0.197 | 0.276 (0.130-0.585) | | 0.001* | 27.1% | 37.3 (10.1–54.1) |
| **Hour of day review message assigned** | | | **0.847** |  |  | **0.714** |  |  |
|  | ***00:00–12:59*** | 1 | – | 1 | | – | 36.7% | 21.6 (2.2–49.2) |
|  | ***13:00–23:59*** | 0.991 (0.903–1.087) | 0.847 | 0.973 (0.840-1.127) | | 0.714 | 34.3% | 22.1 (3.1–69.1) |
| **Time from prescription generated to message assigned** | | | **<0.001*** |  |  | **0.001*** |  |  |
|  | ***< 12 hours*** | 1 | – | 1 | | – | 31.2% | 21.6 (3.1–44.2) |
|  | ***12–23:59 hours*** | 1.335 (1.186–1.503) | <0.001* | 1.036 (0.856-1.254) | | 0.719 | 39.5% | 19.0 (1.9–45.6) |
|  | ***1–6 days*** | 1.241 (1.097–1.404) | 0.001* | 0.836 (0.686-1.019) | | 0.076 | 36.8% | 22.7 (2.2–69.8) |
|  | ***7+ days*** | 1.099 (0.896–1.347) | 0.364 | 0.559 (0.402-0.777) | | 0.001* | 32.7% | 40.3 (3.4–139.4) |

**Significant at p<0.05*

*Results from GEEs accounting for all factors in Table 1 with the exception of Prescription Factor: ‘Prescription status’ which was excluded from the analysis of action and time to action as this can be considered an outcome.*

*ACTION: Categories with zero counts (BNF category ‘Other’ and Mode ‘As required’ and ‘Once-only’) were excluded from the analysis.*

*TIME TO ACTION: Categories with zero counts (Communication theme: ‘Contraindication’, ‘Drug Interaction’; ‘Drug Selection’; ‘Omission’ ‘Other’ and ‘Supporting Information’ were excluded from the analysis.*
