# Supplementary material for: Pharmacist-Physician Communications in a Highly Computerised Hospital: Sign-Off and Action of Electronic Review Messages
Source: PLoS One. 2016 Aug 9;11(8):e0160075. doi: 10.1371/journal.pone.0160075 (PMC4978401; doi:10.1371/journal.pone.0160075)
Supplement: S3 Appendix — (DOCX) [file pone.0160075.s003.docx]

**S3 Appendix:** Results of Generalized Estimating Equation (GEE) for message factors.

**Table A: GEE results for message factors for sign-off rates and time to sign-off ≤ 48 hours**

|  | | **GEE of Sign-off Rates** | | **GEE of Time to Sign-off ≤ 48 hours** | | | **% of Messages** | **Hours to Sign-off**  **(Median, Range)** |
| --- | --- | --- | --- | --- | --- | --- | --- | --- |
|  | | ***Odds Ratio (95% CI)*** | ***p-value*** | ***Odds Ratio (CI)*** | | ***p-value*** | **Signed-off** |  |
| **Grade of the pharmacist** | | | **0.010*** |  | | **0.368** |  |  |
|  | **6** | 1 | – | 1 | | – | 46.4% | 25.5 (2.1–94.3) |
|  | **7** | 0.980 (0.925–1.039) | 0.506 | 1.065 (0.972–1.167) | | 0.178 | 46.7% | 22.4 (2.1–71.3) |
|  | **8** | 0.899 (0.835–0.967) | 0.004* | 1.068 (0.943–1.209) | | 0.300 | 45.9% | 23.4 (2.4–71.8) |
| **Message assigned to a high-risk medicine** | | | **<0.001*** |  |  | **0.713** |  |  |
|  | **No** | 1 | – | 1 | | – | 47.8% | 23.1 (2.1–73.4) |
|  | **Yes** | 0.841 (0.789–0.895) | <0.001* | 0.982 (0.890–1.083) | | 0.713 | 43.0% | 24.1 (2.7–75.4) |
| **Message relates to medicines reconciliation** | | | **0.004*** |  |  | **<0.001*** |  |  |
|  | ***No*** | 1 | – | 1 | | – | 43.1% | 23.3 (2.2–73.5) |
|  | ***Yes*** | 1.082 (1.025–1.142) | 0.004* | 1.210 (1.110–1.319) | | <0.001* | 50.7% | 25.1 (2.9–96.5) |
| **Communication theme** | | | **<0.001*** |  | | **<0.001*** |  |  |
|  | **Dose/Frequency** | 1 |  | 1 | | – | 54.2% | 23.2 (2.4–71.7) |
|  | **Contraindication** | 0.498 (0.439–0.566) | <0.001* | 0.721 (0.579–0.899) | | 0.004* | 33.5% | 40.7 (2.9–120.0) |
|  | **Drug Form/Route** | 0.640 (0.581–0.705) | <0.001* | 1.139 (0.969–1.339) | | 0.116 | 44.0% | 22.6 (1.7–71.9) |
|  | **Drug Interaction** | 0.733 (0.633–0.850) | <0.001* | 0.989 (0.777–1.261) | | 0.932 | 46.6% | 27.0 (2.9–120.2) |
|  | **Drug Selection** | 0.537 (0.494–0.584) | <0.001* | 1.119 (0.969–1.291) | | 0.126 | 35.6% | 23.2 (2.2–72.1) |
|  | **Drug Use/Admin** | 0.604 (0.562–0.649) | <0.001* | 0.863 (0.764–0.974) | | 0.017* | 39.4% | 23.6 (1.7–72.3) |
|  | **Logistics** | 0.560 (0.471–0.665) | <0.001* | 0.957 (0.717–1.278) | | 0.767 | 37.1% | 27.0 (3.7–89.7) |
|  | **Omission** | 0.820 (0.764–0.880) | <0.001* | 0.043 (0.938–1.159) | | 0.437 | 50.1% | 21.7 (2.0–66.8) |
|  | **Other** | 0.864 (0.733–1.018) | 0.080 | 0.566 (0.418–0.766) | | <0.001* | 39.2% | 24.9 (2.7–117.9) |
|  | **Supporting Info** | 0.801 (0.755–0.850) | <0.001* | 0.956 (0.872–1.048) | | 0.339 | 48.4% | 23.8 (2.1–92.9) |
| **Profession** | | | – |  | | **0.001*** |  |  |
|  | **Pharmacist** | – | – | 1 | | – | – | 23.7 (0.7–91.7) |
|  | **Consultant** | – | – | 1.203 (1.020–1.420) | | 0.028* | – | 22.2 (3.1–67.9) |
|  | **Junior** | – | – | 1.159 (1.061–1.267) | | 0.001* | – | 22.9 (2.7–70.9) |
|  | **SPR/NMP** | – | – | 0.976 (0.867–1.099) | | 0.690 | – | 24.3 (4.2–78.2) |

**Significant at p<0.05*

*Results from GEEs accounting for all factors in Table 1. SIGN-OFF: Profession of person signing off the message was excluded from the analysis since the profession of unsigned messages is not possible to determine.*

**Table B: GEE results for message factors for action rates and time taken to action ≤ 24 hours**

|  | | **GEE of Action Rates** | | **GEE of Time to Action ≤ 24 hours** | | | **% of Messages** | **Hours to Action**  **(Median, Range)** |
| --- | --- | --- | --- | --- | --- | --- | --- | --- |
|  | | ***Odds Ratio (95% CI)*** | ***p-value*** | ***Odds Ratio (CI)*** | | ***p-value*** | **Actioned** |  |
| **Grade of the pharmacist** | | | **<0.001*** |  |  | **0.002*** |  |  |
|  | **6** | 1 | – | 1 | | – | 31.9% | 25.2 (3.8–78.1) |
|  | **7** | 1.203 (1.061–1.365) | 0.004* | 1.408 (1.167-1.698) | | <0.001* | 38.1% | 20.4 (2.2–47.5) |
|  | **8** | 1.379 (1.182–1.607) | <0.001* | 1.293 (1.013-1.651) | | 0.039* | 35.1% | 20.2 (1.7–51.4) |
| **Message assigned to a high-risk medicine** | | | **<0.001*** |  |  | **0.707** |  |  |
|  | **No** | 1 | – | 1 | | – | 36.4% | 22.1 (2.4–57.1) |
|  | **Yes** | 0.848 (0.745–0.964) | <0.001* | 1.040 (0.846-1.279) | | 0.707 | 33.9% | 20.4 (1.9–48.2) |
| **Message relates to medicines reconciliation** | | | **0.004*** |  |  | **0.859** |  |  |
|  | ***No*** | 1 | – | 1 | | – | 30.3% | 24.7 (3.4-75.5) |
|  | ***Yes*** | 1.278 (1.144–1.428) | 0.004* | 1.016 (0.852-1.212) | | 0.859 | 42.5% | 23.1 (3.2-68.3) |
| **Communication theme** | | | **<0.001*** |  | | **0.581** |  |  |
|  | **Dose/Frequency** | 1 |  | 1 | | – | 36.4% | 21.6 (2.4–51.6) |
|  | **Drug Form/Route** | 1.099 (0.941–1.285) | <0.001* | 0.992 (0.775-1.268) | | 0.992 | 33.9% | 22.8 (1.4–72.0) |
|  | **Drug Use/Admin** | 0.680 (0.475–0.973) | <0.001* | 0.946 (0.551-1.623) | | 0.839 | 25.2% | 24.2 (3.5–52.7) |
|  | **Logistics** | 1.936 (0.985–3.807) | <0.001* | 0.453 (0.148-1.386) | | 0.165 | 24.5% | 51.4 (22.6–96.5) |

**Significant at p<0.05*

*Results from GEEs accounting for all factors in Table 1 with the exception of Prescription Factor: ‘Prescription status’ which was excluded from the analysis of action and time to action as this can be considered an outcome.*

*ACTION: Categories with zero counts (BNF category ‘Other’ and Mode ‘As required’ and ‘Once-only’) were excluded from the analysis.*

*TIME TO ACTION: Categories with zero counts (Communication theme: ‘Contraindication’, ‘Drug Interaction’; ‘Drug Selection’; ‘Omission’ ‘Other’ and ‘Supporting Information’ were excluded from the analysis.*
