# Supplementary material for: Pharmacist-Physician Communications in a Highly Computerised Hospital: Sign-Off and Action of Electronic Review Messages
Source: PLoS One. 2016 Aug 9;11(8):e0160075. doi: 10.1371/journal.pone.0160075 (PMC4978401; doi:10.1371/journal.pone.0160075)
Supplement: S4 Appendix — (DOCX) [file pone.0160075.s004.docx]

**S4 Appendix:** Results of Generalized Estimating Equation (GEE) for prescription factors.

**Table A: GEE results for prescription factors for sign-off rates and time to sign-off ≤ 48 hours**

|  | | **GEE of Sign-off Rates** | | **GEE of Time to Sign-off ≤ 48 hours** | | | **% Messages** | **Hours to Sign-off**  **(Median, Range)** |
| --- | --- | --- | --- | --- | --- | --- | --- | --- |
|  | | ***Odds Ratio (95% CI)*** | ***p-value*** | ***Odds Ratio (CI)*** | | ***p-value*** | **Signed-off** |  |
| **Speciality** | | | **<0.001*** |  |  | **<0.001*** |  |  |
|  | Medical Admissions | 1 | – | 1 | | – | 52.5% | 20.5 (1.7–48.2) |
|  | Critical Care and Burns | 2.038 (1.796–2.314) | <0.001* | 0.954 (0.786–1.157) | | 0.630 | 63.0% | 23.9 (3.0–72.4) |
|  | General Medicine | 1.111 (1.024–1.205) | 0.011* | 0.775 (0.681–0.883) | | <0.001* | 49.2% | 24.9 (2.4–94.5) |
|  | General Surgery | 0.558 (0.498–0.625) | <0.001* | 0.807 (0.664–0.981) | | 0.031* | 33.3% | 23.0 (1.4–76.6) |
|  | Medical Specialities | 0.840 (0.780–0.906) | <0.001* | 0.809 (0.714–0.917) | | 0.001* | 41.5% | 23.2 (2.2–72.4) |
|  | Surgical Specialities | 0.837 (0.763–0.919) | <0.001* | 0.735 (0.630–0.857) | | <0.001* | 41.9% | 23.9 (2.5–76.9) |
|  | TNO | 1.090 (0.970–1.226) | 0.147 | 0.419 (0.349–0.502) | | <0.001* | 47.0% | 51.3 (19.3–167.5) |
| **BNF category** | | | **<0.001*** |  |  | **<0.001*** |  |  |
|  | CVS | 1 |  | 1 | |  | 51.3% | 24.8 (3.0–95.7) |
|  | CNS | 0.955 (0.891–1.023) | 0.189 | 1.256 (1.131–0.884) | | <0.001* | 44.4% | 23.7 (2.3–78.1) |
|  | Endocrine | 0.993 (0.904–1.091) | 0.883 | 1.323 (1.143–0.875) | | <0.001* | 51.0% | 21.4 (1.6–65.5) |
|  | EEEO | 0.765 (0.650–0.901) | 0.001* | 1.138 (0.880–1.136) | | 0.325 | 43.3% | 22.6 (1.9–75.5) |
|  | GI | 0.894 (0.815–0.981) | 0.018* | 1.032 (0.896–1.116) | | 0.663 | 45.9% | 24.1 (2.5–90.1) |
|  | Infection | 0.991 (0.905–1.086) | 0.852 | 2.062 (1.775–0.563) | | <0.001* | 45.7% | 19.9 (1.3–47.7) |
|  | Malign/Immuno | 1.054 (0.830–1.337) | 0.667 | 1.484 (0.998–1.002) | | 0.051 | 49.2% | 22.6 (2.6–66.6) |
|  | Muscu & Joint | 0.842 (0.724–0.980) | 0.027* | 1.416 (1.100–0.909) | | 0.007* | 42.3% | 23.3 (2.4–68.6) |
|  | Nutrition and blood | 0.796 (0.729–0.868) | <0.001* | 1.022 (0.892–1.121) | | 0.756 | 43.9% | 24.2 (2.7–89.6) |
|  | Obs, Gynae, & Uro | 0.791 (0.656–0.955) | 0.015* | 1.289 (0.953–1.049) | | 0.100 | 45.6% | 22.9 (2.5–66.1) |
|  | Other | 0.511 (0.328–0.796) | 0.003* | 2.521 (0.912–6.969) | | 0.075 | 28.6% | 9.6 (0.5–42.3) |
|  | Respiratory | 0.702 (0.634–0.778) | <0.001* | 1.246 (1.058–1.468) | | 0.008* | 41.4% | 22.3 (1.4–71.2) |
|  | Skin | 0.579 (0.462–0.727) | <0.001* | 1.257 (0.856–1.843) | | 0.243 | 35.2% | 23.8 (2.5–81.7) |
| **Mode of prescription** | | | **<0.001*** |  | | **<0.001*** |  |  |
|  | Regular | 1 |  | 1 | | – | 50.5% | 23.7 (2.5–73.8) |
|  | As Required | 0.538 (0.495–0.586) | <0.001* | 0.813 (0.780–0.935) | | 0.004* | 32.8% | 30.9 (3.7–119.9) |
|  | Once-Only | 0.319 (0.235–0.433) | <0.001* | 11.077 (2.581–47.533) | | 0.001* | 22.2% | 1.8 (0.1–20.2) |
|  | TTO | 0.436 (0.397–0.478) | <0.001* | 2.818 (2.321–3.421) | | <0.001* | 29.5% | 2.1 (0.3–26.2) |
| **Prescription status** | | | **0.005*** |  | | **<0.001*** |  |  |
|  | Continued | 1 | – | 1 | | – | 47.0% | 24.0 (2.5–76.2) |
|  | Deleted | 0.898 (0.832–0.968) | 0.005* | 1.739 (1.520–1.989) | | 0.028* | 42.4% | 5.3 (0.8–44.1) |

**Significant at p<0.05*

*Results from GEEs accounting for all factors in Table 1.*

*SIGN-OFF: Profession of person signing off the message was excluded from the analysis since the profession of unsigned messages is not possible to determine.*

*CVS Cardiovascular; CNS Central Nervous System; Eye, Ear, Nose and Oropharynx; GI Gastrointestinal; Malign/Immuno Malignant Disease and Immunosuppression; Muscu & Joint; Musculoskeletal and Joint Disease; Obs, Gynae, & Uro Obstetrics, gynaecology, and urinary-tract disorders*

**Table B: GEE results for prescription factors for action rates and time taken to action ≤ 24 hours**

|  | | **GEE of Action Rates** | | | **GEE of Time to Action ≤ 24 hours** | | | **% Messages** | **Hours to Action**  **(Median, Range)** |
| --- | --- | --- | --- | --- | --- | --- | --- | --- | --- |
|  | | ***Odds Ratio (95% CI)*** | | ***p-value*** | ***Odds Ratio (CI)*** | | ***p-value*** | **Actioned** |  |
| **Speciality** | | | | **<0.001*** |  |  | **0.093** |  |  |
|  | Medical Admissions | 1 | | – | 1 | | – | 44.9% | 19.9 (2.2–36.0) |
|  | Critical Care and Burns | 0.921 (0.718–1.180) | | 0.515 | 0.831 (0.585-1.180) | | 0.301 | 38.0% | 19.7 (0.7–72.6) |
|  | General Medicine | 1.038 (0.884–1.218) | | 0.651 | 0.832 (0.658-1.052) | | 0.124 | 37.3% | 23.8 (2.2–70.8) |
|  | General Surgery | 0.750 (0.602–0.934) | | 0.010* | 0.767 (0.551-1.068) | | 0.116 | 32.8% | 22.1 (1.9–73.4) |
|  | Medical Specialities | 0.706 (0.610–0.816) | | <0.001* | 0.857 (0.684-1.073) | | 0.179 | 29.1% | 20.4 (2.2–48.0) |
|  | Surgical Specialities | 0.585 (0.483–0.709) | | <0.001* | 0.714 (0.519-0.981) | | 0.038 | 25.9% | 24.0 (4.1–90.2) |
|  | TNO | 1.192 (0.945–1.504) | | 0.139 | 0.598 (0.422-0.848) | | 0.004 | 38.1% | 28.3 (7.2–110.9) |
| **BNF category** | | | | **<0.001*** |  |  | **0.001*** |  |  |
|  | CVS |  | 1 |  | 1 | |  | 37.5% | 23.0 (3.4–68.8) |
|  | CNS | 1.162 (1.024–1.320) | | 0.020* | 1.205 (0.987-1.471) | | 0.067 | 36.2% | 21.4 (1.9–54.5) |
|  | Endocrine | 1.132 (0.944–1.356) | | 0.180 | 1.158 (0.879-1.527) | | 0.297 | 41.8% | 21.7 (2.6–48.1) |
|  | EEEO | 0.872 (0.646–1.176) | | 0.369 | 0.806 (0.499-1.302) | | 0.378 | 34.4% | 24.5 (4.1–97.4) |
|  | GI | 0.771 (0.648–0.917) | | 0.003* | 0.941 (0.709-1.249) | | 0.674 | 26.1% | 23.2 (2.6–75.7) |
|  | Infection | 1.246 (1.048–1.482) | | 0.013* | 2.055 (1.546-2.732) | | <0.001* | 37.8% | 6.0 (1.2–26.4) |
|  | Malign/Immuno | 0.918 (0.555–1.519) | | 0.740 | 0.958 (0.442-2.075) | | 0.913 | 34.6% | 23.4 (2.3–51.0) |
|  | Muscu & Joint | 1.204 (0.888–1.632) | | 0.232 | 1.142 (0.713-1.830) | | 0.580 | 38.6% | 22.0 (2.2–48.2) |
|  | Nutrition and blood | 0.815 (0.677–0.981) | | 0.030* | 1.116 (0.830-1.500) | | 0.467 | 36.2% | 21.4 (3.4–51.8) |
|  | Obs, Gynae, & Uro | 0.892 (0.603–1.320) | | 0.568 | 0.921 (0.504-1.682) | | 0.788 | 36.9% | 23.4 (2.4–44.4) |
|  | Respiratory | 0.906 (0.734–1.119) | | 0.358 | 0.893 (0.645-1.238) | | 0.498 | 36.7% | 24.0 (2.4–69.6) |
|  | Skin | 0.546 (0.297–1.005) | | 0.052 | 1.493 (0.495-4.501) | | 0.477 | 19.5% | 19.2 (2.2–54.0) |
| **Mode of prescription** | | | | **<0.001*** |  | | **0.468** |  |  |
|  | Regular | 1 | |  | 1 | | – | 41.50% | 21.6 (2.2–51.6) |
|  | As Required | 0.374 (0.317–0.442) | | <0.001* | 0.896 (0.666-1.206) | | 0.468 | 18.60% | 24.2 (2.2–90.2) |

**Significant at p<0.05*

*Results from GEEs accounting for all factors in Table 1 with the exception of Prescription Factor: ‘Prescription status’ which was excluded from the analysis of action and time to action as this can be considered an outcome.*

*ACTION: Categories with zero counts (BNF category ‘Other’ and Mode ‘As required’ and ‘Once-only’) were excluded from the analysis.*

*TIME TO ACTION: Categories with zero counts (Communication theme: ‘Contraindication’, ‘Drug Interaction’; ‘Drug Selection’; ‘Omission’ ‘Other’ and ‘Supporting Information’ were excluded from the analysis.*

*CVS Cardiovascular; CNS Central Nervous System; Eye, Ear, Nose and Oropharynx; GI Gastrointestinal; Malign/Immuno Malignant Disease and Immunosuppression; Muscu & Joint; Musculoskeletal and Joint Disease; Obs, Gynae, & Uro Obstetrics, gynaecology, and urinary-tract disorders.*
